# Myocardial protection with Glucose-Insulin-Potassium infusion during adult cardiac surgery

**DOI:** 10.12669/pjms.332.12414

**Published:** 2017

**Authors:** Suhail Ahmad, Rana Altaf Ahmad, Bilal Ahsan Qureshi, Mirza Ahmad Raza Baig

**Affiliations:** 1 Dr. Suhail Ahmad, DA, MCPS, FCPS, M. Sc. Pain Medicine, Associate Professor of Anesthesia and Critical Care, CPE Institute of Cardiology Multan, Pakistan; 2 Dr. Rana Altaf Ahmad, DA, FCPS, M. Sc. Pain Medicine, Professor of Anesthesia and Critical Care, Executive Director, CPE Institute of Cardiology Multan, Pakistan; 3 Dr. Bilal Ahsan Qureshi, Associate Professor of Cardiology, CPE Institute of Cardiology Multan, Pakistan; 4 Mirza Ahmad Raza Baig, B. Sc. Hons CPT, Clinical Perfusionist, CPE Institute of Cardiology Multan, Pakistan

**Keywords:** Coronary artery bypass grafting, Glucose-insulin-potassium (GIK) solution, Myocardial protection

## Abstract

**Background & Objective:**

Recent meta-analysis reports have called for more randomized trials to evaluate the effectiveness of GIK solution in patients of cardiac surgery. So this study was conducted to evaluate the effectiveness of Glucose-insulin-potassium (GIK) solutions in non-diabetic patients undergoing coronary artery bypass grafting.

**Methods:**

A total number of one hundred and sixty (160) patients were randomized into two equal groups; GIK Group and non-GIK group. In GIK group, 5% dextrose containing 70 IU/L regular insulin and 70 meq/L of potassium was administered. The infusion was started at a rate of 30 ml/hour after induction of anesthesia and before the start of cardiopulmonary bypass. The infusion was started again after removal of aortic cross clamp and was continued for six hours after the operation.

**Results:**

In early post-operative period, peak CKMB levels were high in non-GIK group 48.50±19.79 IU/L versus 33.40±14.69 IU/L in GIK group (p-value <0.001). There was no statistically significant difference in requirements of inotropic support between the groups. The mean duration of inotropic support in GIK group was only 5.50±6.88 hours in GIK group and 8.64±7.74 hours in non-GIK group (p-value 0.008). Mean ventilation time in GIK group was 5.06±2.39 hours versus 6.55±3.58 hours in non-GIK group (p-value 0.002). Similarly, ICU stay period was also shorter in GIK group (p-value 0.01). We did not found any detrimental effect of GIK infusion on non-cardiac complications e.g. renal, pulmonary and neurologic complications.

**Conclusion:**

Glucose-insulin-potassium (GIK) infusion has a beneficial role in myocardial protection and is associated with better post-operative outcomes without increasing the risk of non-cardiac complications.

## INTRODUCTION

Use of glucose insulin and potassium (GIK) solution for myocardial protection was 1^st^ time introduced by Sodi-Pollares and colleagues. They used this solution in patients with acute myocardial infarction and concluded that GIK solution limited electrocardiographic changes in these patients.^1^ Several studies have shown reduced morbidity and mortality in patients of acute MI with the use of GIK solution.^2^,^3^ A meta-analysis report calculated that in-hospital mortality is reduced by 28% in patients of acute MI.^4^ Early Studies in isolated hearts have concluded that GIK solution infusion is associated with reduced infarct size and improved ventricular function.^5^,^6^

The role of GIK solution in cardiac surgery patients has also been investigated and studies have proven its efficacy in reducing mortality and improvement in post-operative morbidity.^7^-^10^ During coronary artery bypass grafting (CABG), the myocardium is subjected to endure the periods of ischemia and reperfusion which may result in post-ischemia contractile dysfunction. That is a major contributor of early and late morbidity and mortality and increased requirement of pharmacologic and mechanical circulatory support.^11^,^12^ Meta-analysis by Bothe et al involving 11 randomized trials concluded that GIK administration associated with improved post-operative contractile function and reduced risk of atrial arrhythmias after cardiac surgery.^13^ Most of these studies were conducted in diabetic patients and few studies have been conducted in non-diabetic patients regarding effectiveness of GIK solution in cardiac surgery patients. In this clinical trial we evaluated the effectiveness of GIK solutions regarding myocardial protection and early post-operative surgery outcomes in non-diabetic patients undergoing coronary artery bypass grafting.

## METHODS

This prospective randomized clinical trial was conducted in three months period from March 2016 to June 2016. A total number of one hundred and sixty (160) patients were randomized into two equal groups. Computer generated random numbers were used to assign patients into study and control group. Group-I (GIK group); in this group of patients glucose insulin and potassium infusion was given during surgery and Group-II (non-GIK group); in these patients only glucose infusion was given during the procedure. Initial approval for use of GIK solution in cardiac surgery patients was taken from the department of academic affairs of CPE institute of cardiology. Informed consent was taken before surgery from every patient. Patients of age more than 30 years who underwent multi-vessel coronary artery bypass grafting were selected. Diabetic patients, patients with pre-operative renal dysfunction (creatinine>1.2 mg/dl), valvular operations, those with previous stroke and emergency CABG were excluded.

Draw randomization was used to assign individuals to study and control groups. For study group patients, GIK solutions was prepared before induction of anesthesia by adding 70 IU of regular insulin and 70 meq of potassium in 5% dextrose of 1000 ml and the infusion was started at a rate of 30 ml/hour. In control group, only 5% dextrose infusion was given at 30 ml/hour. The infusion was started after induction of anesthesia and before the start of cardiopulmonary bypass. The infusion was started again after removal of aortic cross clamp and was continued for six hours after the operation. In non-GIK group only dextrose 5% infusion was given in the same manner as that in GIK group. In this group blood glucose levels were controlled using following protocol; RBS 80 to <200=no need for insulin, RBS 200 to 300= 5 units of regular insulin, RBS 300-400=7 units regular insulin, 400-500= 10 units regular insulin given subcutaneously.

Coronary artery grafting was done after establishing standard cardiopulmonary bypass. After clamping the aorta, cold blood cardioplegia was used to arrest the heart and was repeated after completion of each distal graft. Left internal mammary artery was used to vascularize left anterior descending artery in all patients.

Serum potassium and glucose levels were measured before staring the GIK infusion and after every two hours of unclamping the aorta. Standard 12 lead electrocardiogram was taken on 1^st^ and 2^nd^ day after surgery. Post-op CKMB levels were measured after 6, 12 and 24 hours and peak levels were noted. Appearance of Q wave and ST elevation > 1 mm and increase in CK-MB levels more than 125 IU was recorded as perioperative myocardial infarction. Requirement of inotropic support on weaning and in immediate period after cardiopulmonary bypass was noted.

The following criteria were used to classify inotropes:

- **Mild** = Dobutamine<5µg.kg^-1^min^-1^, epinephrine or nor epinephrine < 0.06 µg.kg^-1^min^-1^,
- **Moderate**= Dobutamine 5-10 µg.kg^-1^min^-1^ epinephrine or norepinephrine 0.06-1.0 µg.kg^-1^min^-1^
- **High dose**= Dobutamine> 10 µg.kg^-1^min^-1^, epinephrine or norepinephrine >1 µg.kg^-1^min^-1^.

The primary endpoints of this study were; increase in post-op CKMB levels, intensity and duration of inotropic support and mechanical ventilation time. And non-cardiac complications such as renal, pulmonary and neurologic complications were secondary endpoints of this study.

The criteria to define pulmonary and renal complications in CPEIC cardiac surgery department is already defined in the study of Sher-i-Murtaza et al.^14^ A two fold increase in serum creatinine levels from pre-op baseline value, or need for dialysis after surgery was labelled as renal complication. Occurrence of post-operative pleural effusion, acute respiratory distress syndrome or pneumothorax requiring either parecentesis or chest tube insertion were labelled as pulmonary complications. Occurrence of transient ischemic attacks, transient local paralysis, permanent local paralysis and permanent brain death were labelled as neurologic complications.

Statistical analysis was done through SPSS v23 software. Quantitative variables were compared using t-test and qualitative variables through chi-square test. P-value <0.05 was taken as cut of value for significant difference.

## RESULTS

**Fig.1:**
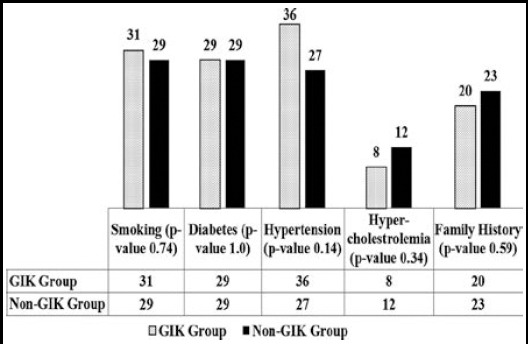
Comparison of risk factors of Ischemic Heart Disease.

There was no significant difference between the mean age and gender of patients in the two groups. Severity of coronary artery disease and pre-operative ejection fraction was not different between the GIK and non-GIK groups. Frequency of risk factors of coronary artery disease was also same in two groups (Fig.1). Regarding operative variables the mean number of grafts implanted, bypass time and cross-clamp time was also not significantly different between two groups (Table-I).

**Table-I:** Patient’s baseline and operative data.

	*GIK Group (n=80)*	*Non-GIK Group (n=80)*	*P-value*
Age of Patients (Y)	55.01±7.69	54.16±9.65	0.53
Male/Female (N)	69/11	72/8	0.46
***Severity of Coronary artery Disease (%)***		
Single vessel disease	0 (0.0)	2 (2.5)	0.32
Two vessel disease	36 (45.0)	32 (40.0)
Triple vessel disease	44 (55.0)	46 (57.5)
Ejection Fraction	53.12±9.13	52.06±9.83	0.48
EURO score	1.13±1.26	1.03±1.13	0.65
Number of grafts	3.0±0.50	2.9±0.84	0.43
Bypass time (minutes)	110.56±26.00	105.82±25.40	0.24
Clamp time (minutes)	64.19±16.73	62.15±17.6	0.45

In early post-operative period, peak CKMB levels were high in non-GIK group 48.50±19.79 IU/L versus 33.40±14.69 IU/L in GIK group (p-value <0.001). There was no statistically significant difference in requirements of inotropic support between the groups but the duration of inotropic support was longer in non-GIK group as compared to GIK group. Ventilator support period was also prolonged in GIK non-group (p-value 0.002). The stay of patients in ICU and hospital was also longer in non-GIK group. There was no difference in non-cardiac complications between the groups. There was no-in hospital mortality (Table-II).

**Table-II:** Early Post-operative Data.

	*GIK Group (n=80)*	*Non-GIK Group (n=80)*	*P-value*
Peak CKMB levels (IU/L)	33.40±14.69	48.50±19.79	<0.001
Peri-operative MI (%)	0 (0.0)	1 (1.3)	0.32
***Requirement of Inotropic Support (%)***		
Mild	41 (51.2)	36 (45.0)	0.81
Moderate	21 (26.3)	31 (38.8)
High dose	3 (3.8)	5 (6.3)
Duration of Inotropic Support (hours)	5.50±6.88	8.64±7.74	0.008
Total Ventilation time (hours)	5.06±2.39	6.55±3.58	0.002
ICU stay (hours)	35.29±11.46	43.48±26.28	0.01
Hospital Stay (Days)	7.00±1.84	7.63±3.19	0.14
***Non-cardiac Complications (%)***		
Neurologic	2 (2.5)	1 (1.3)	0.56
Pulmonary	3 (3.2)	2 (2.5)	0.65
Renal	----	-----	----

## DISCUSSION

For non-ischemic myocardial tissue the main energy source are free fatty acids that provide 60-70% of total myocardial demands. During ischemia these fatty acids are dangerous because these increase O_2_consumption, inhibit glucose consumption and predispose myocardium to arrhythmias with increased formation of O_2_ free radicals.^15^ So provision of exogenous glucose substrate may prove beneficial for myocardium during ischemia.^15^ GIK infusion can lead to metabolic modulation during ischemia & reperfusion e.g. reduced free fatty acid oxidation, improved glucose oxidation, increase in glycolysis, increase in O_2_ uptake for ATP production and its utilization for better myocardial contractility.^16^,^17^ Moreover GIK also plays a role in insulin signaled K-ATP channels activation. These channels are an important mediators of ischemic pre-conditioning that is a powerful protector against ischemia reperfusion injury, MI and apoptosis.^18^,^19^ Insulin plays a key role in the regulation of K-ATP channels by increasing the open state probability and by decreasing their sensitivity to ATP.^20^ Moreover GIK infusion also reported to have vasodilator and inotropic properties.^15^,^21^

Jovic et al found significantly positive role of GIK solution in cardiac surgery patients and concluded that any amount of combination of glucose and insulin concentration can bring this protective effect.^22^ Another large study concluded that GIK infusion in cardiac surgery patients is associated with better cardiovascular performance, less requirement of inotropes and reduced myocardial injury in early postoperative period without increasing the risk of non-cardiac complications.^23^ Recent meta-analysis has concluded that peri-operative GIK infusion improve cardiac performance and myocardial injury in CABG patients.^24^ Another meta-analysis has reported similar effects.^25^ Our study have shown similar results as compared to these studies.

In our study, we found significantly positive effects of GIK infusion on release of CKMB enzymes after surgery, inotropic support and intensive care unit (ICU) stay period. In our study, peak post-surgery CKMB levels were 33.40±14.69 IU/L in GIK group and 48.50±19.79 IU/L in non-GIK group (p-value <0.001). The mean duration of inotropic support in GIK group was only 5.50±6.88 hours in GIK group and 8.64±7.74 hours in non-GIK group (p-value 0.008). The patients in GIK group was early extubated as compared to the non-GIK group. Mean ventilation time in GIK group was 5.06±2.39 hours versus 6.55±3.58 hours in non-GIK group (p-value 0.002). Similarly, ICU stay period was also shorter in GIK group as compared to non-GIK group (p-value 0.01). We did not found any detrimental effect of GIK infusion on non-cardiac complications e.g. renal, pulmonary and neurologic complications. So we found significantly positive effects of GIK infusion in coronary artery bypass grafting patients.

## CONCLUSION

Glucose-insulin-potassium (GIK) infusion has a beneficial role in myocardial protection and is associated with better post-operative outcomes without increasing the risk of non-cardiac complications.

### Authors` Contribution

**SA:** Conceived, designed, & wrote the manuscript.

**RAA:** Supervision and editing of the manuscript.

**BAQ:** Did review and helped in data analysis.

**MARB:** Did data collection, compilation and analysis.
